# Addressing Human Variability in Next-Generation Human Health Risk Assessments of Environmental Chemicals

**DOI:** 10.1289/ehp.1205687

**Published:** 2012-10-19

**Authors:** Lauren Zeise, Frederic Y. Bois, Weihsueh A. Chiu, Dale Hattis, Ivan Rusyn, Kathryn Z. Guyton

**Affiliations:** 1 Office of Environmental Health Hazard Assessment, California Environmental Protection Agency, Oakland, California, USA; 2 Institut National de l’Environnement Industriel et des Risques (INERIS), Verneuil en Halatte, France; 3 National Center for Environmental Assessment, U.S. Environmental Protection Agency, Washington, DC, USA; 4 George Perkins Marsh Institute, Clark University, Worcester, Massachusetts, USA; 5 Department of Environmental Sciences and Engineering, University of North Carolina–Chapel Hill, Chapel Hill, North Carolina, USA

**Keywords:** environmental agents, genetics, human health risk assessment, modeling, omics technologies, susceptible populations, variability

## Abstract

Background: Characterizing variability in the extent and nature of responses to environmental exposures is a critical aspect of human health risk assessment.

Objective: Our goal was to explore how next-generation human health risk assessments may better characterize variability in the context of the conceptual framework for the source-to-outcome continuum.

Methods: This review was informed by a National Research Council workshop titled “Biological Factors that Underlie Individual Susceptibility to Environmental Stressors and Their Implications for Decision-Making.” We considered current experimental and *in silico* approaches, and emerging data streams (such as genetically defined human cells lines, genetically diverse rodent models, human omic profiling, and genome-wide association studies) that are providing new types of information and models relevant for assessing interindividual variability for application to human health risk assessments of environmental chemicals.

Discussion: One challenge for characterizing variability is the wide range of sources of inherent biological variability (e.g., genetic and epigenetic variants) among individuals. A second challenge is that each particular pair of health outcomes and chemical exposures involves combinations of these sources, which may be further compounded by extrinsic factors (e.g., diet, psychosocial stressors, other exogenous chemical exposures). A third challenge is that different decision contexts present distinct needs regarding the identification—and extent of characterization—of interindividual variability in the human population.

Conclusions: Despite these inherent challenges, opportunities exist to incorporate evidence from emerging data streams for addressing interindividual variability in a range of decision-making contexts.

Human variability underlies differences in the degrees and ways in which people respond to environmental chemicals, and addressing these differences is a key consideration in human health risk assessments for chemicals [Guyton et al. 2009; Hattis et al. 2002; National Research Council (NRC) 2009]. A large array of possible health outcomes is of concern for such assessments, and many sources of variation can influence the severity and frequency of the adverse effects at different exposure levels. These sources may be intrinsic (e.g., heritable traits, life stage, aging), or extrinsic, exogenous, and acquired (e.g., background health conditions, co-occurring chemical exposures, food and nutrition status, psychosocial stressors). Interactions between inherent and extrinsic factors create the large range of biological variation exhibited in response to a chemical exposure (NRC 2009). Given that biological variability in susceptibility is context-dependent, so too is the extent to which it needs to be described and quantified to inform any particular environmental decision. The salience of variability information for specific choices is affected by the range of available risk management options; the regulatory authority; the available time, resources, and expertise to collect data and conduct analyses; and stakeholder concerns.

Over the past decade, efforts to systematically “map” human variability have expanded dramatically, focusing mainly on genetic variation (Schadt and Björkegren 2012). In addition to genetic differences, omics studies have examined the impact of epigenetic, transcriptomic, proteomic, and metabolomic variation on disease susceptibility, prognosis, or options for pharmacotherapy (Chen et al. 2008; Emilsson et al. 2008; Illig et al. 2010; Manolio 2010; Schadt 2009). Tailored chemotherapy treatment based on patient (Phillips and Mallal 2010) or tumor (La Thangue and Kerr 2011) genetics is an example of a significant success in applying such discoveries; however, for many diseases, the substantial nongenetic variation in disease or treatment outcomes has limited their utility. Thus, the characterization of the broad set of environmental factors, including those related to chemical exposures, that may contribute to disease is directly relevant to both personalized medicine and environmental health protection (Khoury et al. 2011).

In this review, we explore how next-generation (“NexGen”) human health risk assessments of chemicals might take advantage of novel data to better characterize and quantify variability in susceptibility, by using and expanding upon current analytical methods. We begin by describing biological variability through the conceptual framework of the source-to-outcome continuum. Next, the utility of that framework is illustrated in a review of current approaches to describing variability in susceptibility in human health risk assessments. Then, emerging data streams that may be informative in characterizing human variability in susceptibility are described. Finally, we consider the opportunities, challenges and methods for using emerging data to help assess interindividual variability in responses to environmental chemicals across different decision contexts.

## Susceptibility as a Function of the Source-to-Outcome Continuum and Biological Variability

**Figure 1:**
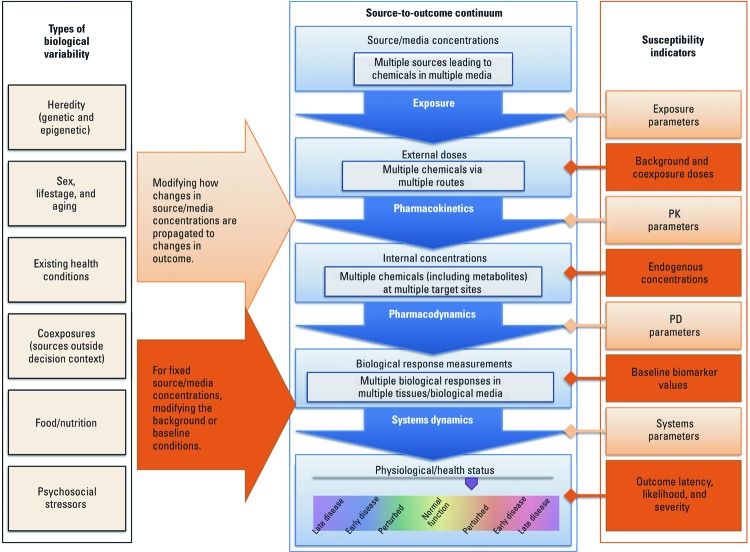
Framework illustrating how susceptibility arises from variability. Multiple types of biological variability intersect with the source-to-outcome continuum, either by modifying how changes to source/media concentrations propagate through to health outcomes or by modifying the baseline conditions along the continuum. The aggregate result of all these modifications is variability in how a risk management decision impacts individual health outcomes. The parameters and initial conditions along the source-to-outcome continuum serve as indicators of differential susceptibility, some of which are more or less influential to the overall outcome (see Figure 2).

The “source-to-outcome continuum” [U.S. Environmental Protection Agency (EPA) 2007; NRC 2007] is a conceptual framework for human health risk assessment of environmental chemicals in which changes in the sources of chemicals in the environment are further propagated within the individual through a series of biological and physiological steps that may ultimately manifest as an adverse health outcome (Figure 1):

*Source/media concentrations* are measures of the chemical, which may change under specific risk management options being considered. A given risk management decision may differentially affect media concentrations depending on local conditions.

*External doses* are measures of exposure (e.g., concentration in air × breathing rate per body weight) to or intake (e.g., amount ingested per body weight) of environmental chemicals, and are related to source/media concentrations by exposure pathways. Sources of variability that may confer susceptibility include differences in behaviors, such as breathing rates, water consumption, and dietary habits (e.g., the amount of fish consumed), and, in an occupational context, use of personal protective equipment.

*Internal doses* are the amounts/concentrations of environmental chemicals or their metabolites at the target site(s) of interaction with biological molecules, and are related to external doses by pharmacokinetic (PK) processes. Susceptibility may arise from differences in compartment sizes and composition (e.g., fat concentration in plasma, which rises during pregnancy) (Roy et al. 1994), as well as differences in the rates of uptake (e.g., fraction absorbed from diet or air), metabolism, elimination, and transport to sites of action (e.g., the blood–brain barrier). Such differences may be due, for example, to genetics (e.g., via polymorphisms in metabolic enzymes, uptake and efflux transporters), other chemical exposures (via metabolic enzyme induction and inhibition), and preexisting health conditions and life stage (e.g., via metabolism and mobilization from tissue storage).

*Biological responses* are measures of biological state (e.g., the concentration of glutathione) altered by interactions with environmental chemicals or their metabolites, and are related to internal doses by pharmacodynamic (PD) processes. Variation leading to differential susceptibility can stem from differences in transport systems, receptors and/or proteins in other toxicity pathways, as well as repair capacity (of, for example, DNA), which in turn are affected by intrinsic and extrinsic factors such as genetics and life stage.

*Physiological/health status* reflects the overall state, structure, or function of the organism and is related to biological responses through systems dynamics, the underlying physiological status of the host to which the chemical-specific perturbation is added. Examples include maintenance and adaptation processes (associated with preexisting health conditions, sex hormone levels, for example), and the accumulation of damage events from past exposures (e.g., loss of alveolar septa from past cigarette smoke exposure). Variation in these can confer susceptibility by altering the likelihood of progression from normal function to mild perturbations, early disease, and late disease. Systems dynamics describes the propagation of biological perturbations regardless of whether they are due to chemical exposure, thus distinguishing it from pharmacodynamics, which describes how chemical exposure causes biological perturbations.

**Figure 2:**
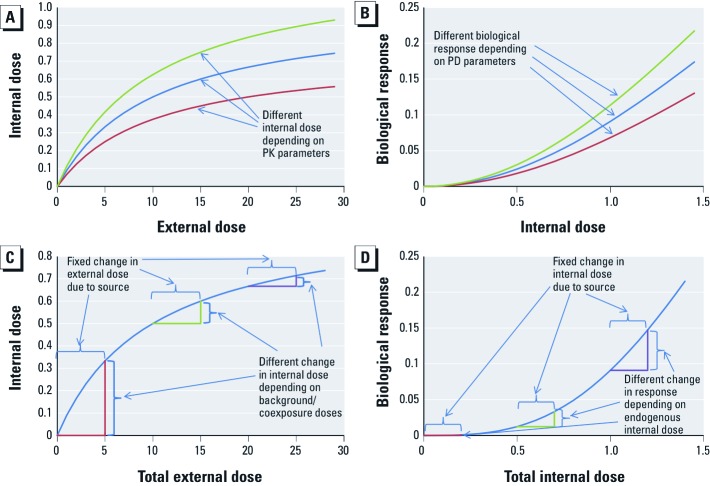
Effects of variability in PK (*A*), PD (*B*), background/coexposures (*C*), and endogenous concentrations (*D*). In (*A*) and (*B*), individuals differ in PK or PD parameters. In (*C*) and (*D*), individuals have different initial baseline conditions (e.g., exposure to sources outside of the risk management decision context; endogenously produced compounds).

Figure 2 illustrates the distinct effects of different sources of variability on external dose, internal dose, or biological response. The first category of biological variability is indicated by differences in the parameters governing the relationship of one measureable quantity to the next (e.g., external to internal dose, and internal dose to biological response) (Figure 2A,B). In addition, there may be biological variability in the initial conditions for each measureable quantity, as well as the contribution from the source of environmental chemical exposure under consideration for risk management (Figure 2C,D). For example, increases in background exposure to the same or a different chemical(s) may result in saturation of metabolic activation and/or clearance processes, or temporary depletion of cofactors involved in detoxification, such as glutathione, resulting in either attenuation or amplification of the effect of additional increments of chemical exposure on internal dose (Figure 2C). Nonetheless, a biological response with a low background level may be much less altered by additional exposure than one with a high background because of to the cooperativity associated with a relatively higher baseline internal dose (Figure 2D).

## Current Approaches to Addressing Variable Susceptibility

Variability for assumed threshold-like dose–response relationships is currently addressed by applying an “uncertainty” or “adjustment” factor (U.S. EPA 2011). The factor to account for interindividual variability in human population has typically been 1, 3, or 10. In some cases, the factor is further divided to separately account for variation in PK and PD (U.S. EPA 2011; International Programme for Chemical Safety 2001). In this context, PD has included both PD and systems dynamics processes described above and in Figure 1. Data permitting, the PK component can be addressed through physiologically based pharmacokinetic (PBPK) modeling, in which case a factor addressing only PD is applied (U.S. EPA 2011). Occasionally, exposure–effect observations are available for particularly susceptible human populations, such as with ozone and persons with asthma (U.S. EPA 2006), or those sensitive to chronic beryllium disease (U.S. EPA 1998), which allows for a data-driven estimation of the likely impact of interindividual variability on human health risk assessments.

For presumed nonthreshold cancer end points, interindividual variability is not currently addressed when risk is estimated from animal studies, with the exception that for mutagenic compounds exposures occurring early in life are weighted more heavily (by a factor of 10 between birth and 2 years of age, and a factor of 3 between 2 and 16 years of age). Cancer risk for susceptible populations, such as smokers who have been exposed to radon, may be calculated in addition to that for a general population (U.S. EPA 2003). Alternatively, adjustments may be made to address susceptible subgroups, such as the sex-specific effects of 1,3-butadiene (U.S. EPA 2002). There have been calls to formally account for variability in cancer dose response (NRC 2009).

Over the past 30 years, several strategies to characterize (predominantly PK) variability combining mathematical models and statistical distributions have developed in parallel. The first strategy, mostly used for data-rich pharmaceuticals, couples empirical PK models and multilevel (random effect) statistical models to extract *a posteriori* estimates of variability from clinical data on patients or volunteers. This “population PK” approach (Beal and Sheiner 1982) seeks to measure variability and to discover its determinants. The second, the “predictive PK,” approach takes advantage of the predictive capacity of mechanistic models and assigns *a priori* distributions to their parameters (e.g., blood flows, organ volumes). The parameters having biological meaning can be observed through independent experiments, clinical measurements, or surveillance. Table 1 lists some examples of data sources for developing *a priori* parameter distributions. Monte Carlo simulations are used to propagate the distributions from model parameters to model predictions (Portier and Kaplan 1989; Spear and Bois 1994). A third approach, the “Bayesian PBPK” approach, offers a synthesis of the other two, applying mechanism- or chemical-specific parameter variability data from a variety of independent sources while using population observations of relevant biomarkers of internal exposure and effect to further inform parameter variability (Allen et al. 2007; Bernillon and Bois 2000; Hack 2006). Parameter covariance can be modeled by multivariate prior distributions (Burmaster and Murray 1998) or joint posterior distributions obtained by Bayesian multilevel modeling (Bois et al. 1990; Wakefield 1996). A Bayesian PBPK model-based analysis of the population toxicokinetics of trichloroethylene (TCE) and its metabolites in mice, rats, and humans provides a practical example of how a systematic method of simultaneously estimating model parameters and characterizing their uncertainty and variability can be applied to a large database of studies on a chemical with complex toxicokinetics (Chiu et al. 2009).

**Table 1:** Examples of data sources for modeling PK and PD variability.

Example	References
Variability in human phase I and phase II metabolism and renal excretion, including in different age groups–neonates, children, and the elderly	Dorne 2010; Ginsberg et al. 2002, 2004; Hattis et al. 2003
Compilations of genetic polymorphisms of specific metabolic enzyme activities:
Paraoxonase	Ginsberg et al. 2009a
N-Acetyltransferase 1 and 2	Bois et al. 1995; Walker et al. 2009
Glutathione transferases	Ginsberg et al. 2009b
CYP2D6 (cytochrome P450 2D6)	Neafsey et al. 2009b
CYP2E1 (cytochrome P450 2E1)	Neafsey et al. 2009a
ALDH2 (acetaldehyde dehydrogenase 2)	Ginsberg et al. 2009c
Human biomonitoring observations of interindividual differences in biomarkers of exposure (e.g., chemical-protein adducts) or in levels of parent/metabolite	Bois et al. 1996
Variability in physiological parameters for older adults: bodymass, surface area, body mass index, health status	Thompson et al. 2009
Indicators of PD variability		
Human DNA repair enzyme XRCC1	Ginsberg et al. 2011
Human host defense enzymes	Ginsberg et al. 2010
Lung function response to particulate matter	Hattis et al. 2001
Susceptibility to infectious organisms	Hattis 1997

PBPK models have been often used to assess variability on the basis of prior parameter distributions obtained from *in vitro* experiments or the physiological literature (Bois et al. 2010; Jamei et al. 2009) and can include genetic information regarding variability. For example, PBPK models can inform the implications of polymorphisms in metabolism genes (Johanson et al. 1999). The effects of such polymorphisms on PK of environmental toxicants and drugs have been the subject of many empirical studies (reviewed by Ginsberg et al. 2009c, 2010). These polymorphisms are of particular concern for xenobiotics whose metabolic fate or mechanism(s) of action is controlled by a particular enzyme (Ginsberg et al. 2010), and in such cases genetic variability can profoundly influence enzyme function with implications for internal dose (Figure 1). However, because enzymatic pathways with overlapping or redundant function and other pharmacokinetic factors (e.g., blood flow limitation) can also influence metabolic fate (Kedderis 1997), PBPK models are needed to evaluate the implication of genetic polymorphisms in metabolizing enzymes in human health risk assessment (Ginsberg et al. 2010).

The situation is somewhat different for PD and systems toxicology models. The biologically based dose–response models describe apical or intermediate end point responses as a function of PK-defined internal doses (Crump et al. 2010). However, models designed purely from our understanding of the disease process, such as the role of cytotoxicity and regenerative proliferation in carcinogenesis (Luke et al. 2010b), or the effect of dietary iodide and thyroid hormones on the hypothalamic–pituitary–thyroid axis (McLanahan et al. 2008), require further development to reliably predict an adverse outcome from tissue exposure (the last two arrows in Figure 1), or its variability. Understanding a disease process at the pathway level (i.e., PD and systems dynamics components of the source-to-outcome continuum) is in itself not sufficient to define reliable and informative mechanistic models because of great model sensitivity to uncertain inputs. Most such models are based on equations derived from the classical receptor theory (Csajka and Verotta 2006) and focus on PD rather than system dynamics elements of the disease process and do not attempt to model the full process from tissue exposure to disease outcome.

## Emerging Data Streams on Biological Variability

Experimental population-based paradigms to address intrinsic variability in response to exposure comprise multiple levels of biological organization, from molecules to whole bodies. Published examples, reviewed by Rusyn et al. (2010), include animal models and large-scale *in vitro* screening platforms to study population-based genetic determinants. Those studies have also aided in the identification of genetic susceptibility factors that underlie toxicity phenotypes. Complementary to these are genome-wide (Hutter et al. 2012) and exposure-wide (Patel et al. 2010) association studies for assessing human population variability.

*Experimental* in vitro *data on genetic variability.* Human cell lines obtained from genetically diverse subjects and multiple populations (Durbin et al. 2010) hold the promise of providing data for assessing genetic determinants of different components of toxic response. Many recent studies have used human lymphoblastoid cell lines, representative of the genetic diversity in populations of European, African, Asian, and North and South American ancestry, to quantify interindividual and interpopulation variability in response to drugs (Welsh et al. 2009). Dozens of studies published in the past 5 years have profiled the cytotoxicity of single to as many as 30 drugs (mostly chemotherapeutics) in hundreds of cell lines. Diverse applications for such a population-based cell model has been suggested. Drug class–specific signatures of cytotoxicity, which could indicate possible shared mechanisms, have been identified and replicated in both cell lines from different populations and for additional compounds (Watson et al. 2011). Furthermore, such studies may potentially inform the prioritization of chemotherapeutic drugs with a sizable genetic response component for future investigation (Peters et al. 2011) and assist in identifying germline predictors of cancer treatment outcomes (Huang et al. 2011).

The utility of such *in vitro* models to toxicology, especially for exploring the extent and nature of genetic components of interindividual variability in PD and systems dynamics, was recently demonstrated (Lock et al. 2012; O’Shea et al. 2011). Quantitative high-throughput screening (qHTS) produced robust and reproducible data on intracellular levels of adenosine triphosphate and caspase-3/7 activity (i.e., biological response) indicative of general cytotoxicity and activation of apoptosis (i.e., physiological status), with utility for variability assessment as follows. First, standardized and high-quality concentration–response profiling, with reproducibility confirmed by comparison with previous experiments, enables prioritization of chemicals based on interindividual variability in cytotoxicity. Second, genome-wide association analysis of cytotoxicity phenotypes allows exploration of the potential genetic determinants of that variability. Finally, the highly significant associations between basal gene expression variability and chemical-induced toxicity suggest plausible mode-of-action hypotheses for follow-up analyses.

Several extensions of these studies can be envisioned to advance the identification of determinants of genetic susceptibility and variability in toxic response. Opportunities include the testing of additional, and more diverse, chemicals (including major metabolites) and concentrations (to account for lower metabolic capacity of these cells). Other specific end points could also be assessed. Further, these studies could be expanded to include larger panels of lymphoblasts and other cell types from genetically and geographically diverse populations. Development of related assay systems to monitor differences in susceptibility to perturbation of communication between cells (e.g., neurotransmission or differentiation signals) could address other aspects of variability not present in cultures comprising only one kind of cell. The development and use of these and other types of *in vitro* assays would be further informed by quantitative comparisons of the PD interindividual variability measured *in vitro* with observable human pharmacodynamics variability *in vivo*. Candidate chemicals for this comparison would be selected environmental toxicants (such as ozone) and pharmaceuticals that have been tested for responses in appreciable numbers of human subjects at different known exposure levels. The extent of interindividual variability in response that was observed for different chemicals in *in vitro* assays could also be compared with previously collected sets of *in vivo* human PD variability data (Hattis et al. 2002).

*Experimental* in vivo *data.* Several proof-of-concept studies that utilized a “mouse model of the human population” have demonstrated the potential for translation to clinical applications and for addressing both PK and PD components of variability (Guo et al. 2006, 2007; Harrill et al. 2009b; Kleeberger et al. 1997; Prows et al. 1997). For example, the extent and nature of TCE metabolism is an important consideration in relating adverse health effects in rodents to humans. Bradford et al. (2011) measured variability in PK for TCE using a panel of inbred mouse strains, revealing marked differences among individual mice (e.g., a greater than 4-fold difference in peak serum concentrations of TCE metabolites). These experimental data on intraspecies differences in TCE metabolism may be used to calibrate the variability in outputs of PBPK models, and thus inform quantitative assessment of variability in TCE metabolism across species.

With regard to PD variability, genetically diverse mouse strains can be used to understand and predict adverse toxicity in heterogeneous human populations. For example, Harrill et al. (2009a) evaluated the role of genetic factors in susceptibility to acetaminophen-induced liver injury in a panel of inbred mouse strains and two cohorts of human volunteers. The authors identified genes associated with differential susceptibility to toxicity in a preclinical phase. This finding has the potential to focus further toxicogenetics research, overcome the challenges of studies in small human cohorts, and shorten the validation period. The data acquired with this model may be used in analyses of individual risk to toxicants. Furthermore, when combined with omics data collected on an exposed population of individual strains, it may be possible to explore underlying genotype-dependent and -independent toxicity pathways involved in PD response (Bradford et al. 2011; Harrill et al. 2009a).

Experiments such as these afford the opportunity to quantitatively understand the interplay between genetics, PD, and systems dynamics. In addition, genetically defined mouse models may be used to supplement the limited data from human studies to not only discover the genetic determinants of susceptibility and understand the molecular underpinnings of toxicity (Harrill et al. 2009a; Koturbash et al. 2011) but also to develop descriptions of variability for use in dose–response and mechanistic evaluation components of human health risk assessments.

Such rodent systems can also be used to assess the role of epigenetics, as well as its potential interplay with the genetic background, in susceptibility. For example, Koturbash et al. (2011) demonstrated that interstrain differences in susceptibility to 1,3-butadiene–induced genotoxicity may be due to strain-specific epigenetic events that are also part of a PD response.

Practical use of this type of experimental information is possible mainly when the mechanistic pathways to human adverse responses are better established. More general application will also depend on the development of suites of rodent models that more fully represent human diversity in both genetics and other factors, such as age (Hamade et al. 2010). Such studies can, in turn, provide important insights concerning the identity and extent of sources of variability that may arise in the source-to-outcome continuum for a given chemical class, physiologic state, or adverse response.

*Human clinical and observational data.* Genome-wide association studies (GWAS) with disease severity as the phenotypic trait are used to associate genetic loci with risk for complex diseases (Rosenberg et al. 2010). Even though GWAS approaches have uncovered numerous genomic loci that may affect the risk of human disease (Manolio 2010), the identified variants explain only a small proportion of the heritability of most complex diseases (Manolio et al. 2009). Some have suggested that unexplained heritability could be partly due to gene × environment interactions, or complex pathways involving multiple genes and exposures (Schadt and Björkegren 2012).

The GWAS concept is now being applied to identify additional genotype-dependent metabolic phenotypes and to gain insight into nongenetic factors that contribute to the effects of xenobiotics on system dynamics. In animal studies, metabolic phenotype-related quantitative trait loci were shown to be useful in understanding genome × phenotype relationships and how extended genome (microbiome) perturbations may affect disease processes through transgenomic effects (Dumas et al. 2007). In a series of human studies (Gieger et al. 2008; Illig et al. 2010; Suhre et al. 2011), serum collected from two large European cohorts (2,820 individuals in total) was analyzed with nontargeted metabolomics, focusing on endogenous metabolites and covering 60 biochemical pathways. Ratios of metabolites to parent chemical concentrations served as surrogates for enzymatic rate constants. Thirty-seven genes were associated with blood metabolite concentrations and, in some cases, explained a substantial fraction of the variance. Endogenous and xenobiotic metabolites (mostly of drugs) were studied.

Clinical (Brown et al. 2008; Hernandez et al. 2010) and epidemiological (Jia et al. 2011; Wood et al. 2010) studies of acute and chronic effects of ambient air exposures have long had important roles in quantifying human variability in the risks of exposures to widespread toxicants such as ozone and airborne particulates. The addition of GWAS to these established tools has the potential to widen the capability for quantification of effects on susceptibility of many individual genotypic variants that individually have relatively modest effects (Holloway et al. 2012). Establishing the roles of individual pathways in affecting susceptibility via genetic analysis, in turn, has the potential to advance the assessment of effects of other exposures during life that also affect the same pathways. Elucidating these determinants for prominent toxicants, however, requires a very considerable research effort. Nonetheless, this research paradigm provides opportunities to explore variability in adverse responses that is due to physiological states for which *in vitro* and experimental animal models are lacking.

Variability in human response to an agent stems in part from differences in the underlying exposures that contribute to a given disease response prevalence within the population. A person’s internal “chemical environment” may be as important for possible disease associations as exposures to the variety of chemicals in the external environment. Under this “exposome” concept (Wild 2005), exposures include environmental agents and internally generated toxicants produced by the gut flora, inflammation, oxidative stress, lipid peroxidation, infections, and other natural biological processes (Rappaport and Smith 2010).

## Advances in *in Silico* Methods to Address Human Variability

Modeling of variability is expected to be needed for both data-rich and -sparse chemicals. Recent advances in software, publicly available data and ongoing computational activities in biomedical research should facilitate the development and use of the results of this type of modeling.

*Modeling the PK dimension of human variability.* Commercial software products [e.g., by Simcyp (http://www.simcyp.com), Bayer Technology (http://www.pksim.com)] are available to explicitly address variability for pharmaceutical or human health risk assessment applications to, for example, adjust dosing for different target patient populations (Jamei et al. 2009; Willmann et al. 2007). Several of these offer generic PBPK models, applicable to “any” substance; however, their substance-specific parameters have to be obtained from *in vitro* experiments (particularly on metabolism) or quantitative structure–property relationships. The variability of subject-specific physiological parameters can be informed by compiled databases (see above) and literature searches (Bois et al. 2010; Ginsberg et al. 2009c), and could include adjustments or protocols to address limitations in data availability. Quantitative structure–property relationship models or *in vitro* data can also be used to derive substance-specific parameters. These models are being applied in an exploratory fashion in *in vitro*–based assessments (Judson et al. 2011; Rotroff et al. 2010).

Using a Bayesian multilevel population approach, some of the key parameters of these generic models could be calibrated by integrating human observational data with data from lower levels of biological organization. This presents a computational challenge on a chemical-specific basis, because those models are neither particularly parsimonious nor quickly evaluated. Yet an extensive calibration of a complex generic model for a selected number of data-rich environmental or pharmaceutical chemicals could be used as support to develop generic approaches for PK variability treatment in human health risk assessment. For example, generalizations could be made about the extent to which particular enzymes may contribute to overall human PK. Extensions of the approach of Hattis et al. (2002) can also be developed to construct “bottom up” quantitative descriptions of PK variability that can be applied as defaults across classes of chemicals.

*Modeling the PD dimension of human variability.* Semi-empirical PD models can include observed biomarkers of susceptibility as covariates. Such models are increasingly applied in predictive toxicity and human health risk assessment. Environmental epidemiology also routinely models quantal types of biomarker data in logistic regressions. Harmonizing the tools and models of toxicological risk assessment with those of epidemiological risk assessment, and reconciling their data and results, should facilitate the development of better approaches for background and variability descriptions in NexGen human health risk assessments.

*Integrating PK and PD into a systems biology framework.* The link between toxicity pathway and “normal cell physiology” models of systems biology could also be further developed and used as the basis to explore potential ranges of human variability. The potential of publicly accessible and curated biomodel and database repositories will be increasingly exploited as familiarity increases in the risk assessment and risk management communities. Importantly, systems biology models can describe background biological processes and the impact of their perturbation and provide a framework for exploring human variability and identifying susceptible populations for targeted assessment and management efforts. Although they come at the price of tremendous complexity, their development can leverage the considerable ongoing effort by the biomedical and pharmaceutical research community to support applications other than toxicant risk evaluation. Further, because of these large-scale efforts, the necessity of sharing and standardization is well understood in the United States. The systems biology markup language (Hucka et al. 2003), for example, is a high-level language developed explicitly to provide a common intermediate format for representing and exchanging systems biology models. Predictive toxicology will benefit from these developments.

The frontier for both PK and PD is in the integration of the rapidly growing information about metabolic networks, receptors, and their regulation with toxicity pathways. The models so far most amenable to quantitative predictions are differential equation models. PBPK models will likely be merged with systems biology and virtual human models. The boundary between PK and PD actually tends to blur as metabolism becomes more and more integrated into detailed models of toxicity pathways when, for example, modeling enzymatic induction by xenobiotics (Bois 2010; Luke et al. 2010a). The variability of the different components of those models will be directly informed by time series of genomic, proteomic, metabolomic data on the chemical species considered. This may provide a framework for assessing the variability in susceptibility to chemically induced effects as influenced by possible metabolic interactions as well as preexisting disease. In time this may facilitate computing the impact of, for example, single nucleotide polymorphisms on the reaction rates of enzymes and receptors and translating these calculations to estimates of human variability (Mortensen and Euling 2011). Ongoing work on simulations of enzymatic reactions or receptor binding at the atomic level (e.g., the potassium channel pore) shows the way forward for predicting fundamental reaction rates by physical chemistry approaches. Prediction of the quantitative impact of sequence or amino-acid variation on the function of the reactive species involved in systems biology models is coming within reach (Giorgino et al. 2010; Sadiq et al. 2010).

Biologically based PD models, such as the systems biology models of response networks (Schuster 2008), models of toxicity pathway perturbations, and biologically based dose–response models proposed to link biochemical responses to apical effects, clearly hold promise (Csajka and Verotta 2006; Jonsson et al. 2007; Nong et al. 2008) but face challenges similar to those that hampered the use of biologically based cancer models (Bois and Compton-Quintana 1992; Chiu et al. 2010). To explore the extent of human variability in response to toxicant and stressor exposures, the various steps in the relevant causal path need to be modeled quantitatively and on a population basis. A problem is that the quantitative linking of omics biomarkers to risk is missing. For many markers (e.g., of apoptosis, cell division), the linkage to risk is highly uncertain (Woodruff et al. 2008), so the ranges of possible variability may be very large. Further, the ability to reinforce information by linking with the impact of injury on multiple targets is also limited because such links are generally not well understood.

## Implications for NexGen Human Health Risk Assessments

Multiple “tiers” of human health risk assessment needs, requiring different levels of precision, can be envisioned. These include screening-level analyses of multiple chemicals to inform the prioritization of management and enforcement actions across communities, ensuring protection across the population to widespread exposure to legacy contaminants, or identifying subpopulations for which differing risk management options might be applied.

In the lowest (simplest) tier of assessments, evaluations are expected to primarily rely on the results of high- and medium-throughput *in vitro* screening tests in mostly human cell lines, as well as complementary *in silico* predictive methods. The Tox21 collaboration (Collins et al. 2008) is leading the field in exploring how a broad spectrum of *in vitro* assays, many in qHTS format, can be used to screen thousands of environmental chemicals for their potential to disturb biological pathways that may result in human disease (Xia et al. 2008). Such data on toxicologically relevant *in vitro* end points can be used as toxicity-based triggers to assist in decision making (Reif et al. 2010), as predictive surrogates for *in vivo* toxicity (Martin et al. 2010; Zhu et al. 2008), to generate testable hypotheses on the mechanisms of toxicity (Xia et al. 2009), and to develop screening assays based on pathway perturbations. The extent of interindividual variability in toxic response to be estimated from these types of assays can be informed by empirical data and PK/PD models that address multiple factors in the source-to-outcome continuum as described in Figure 1. The genomic component of variability may be partially informed by test data from genetically diverse but well-defined human cell lines, such as from the HapMap (http://hapmap.ncbi.nlm.nih.gov/) and 1000 Genomes (http://www.1000genomes.org/) projects. For example, emerging data based on standardized and high-quality concentration–response profiling can help inform characterizations of the extent of interindividual variability in cytotoxicity. When chemical-specific estimates are lacking, the range of interindividual variability for structurally related compounds may be informative, in a read-across approach. Quantitative data characterizing the range in response (e.g., size and variance) may be integrated with probabilistic default distributions addressing the remaining key sources of interindividual variability. Quantitative estimates of PK variability would be also incorporated. In addition, factors such as life stage and background exposures may be particularly important considerations for approaches accounting for baseline differences in the spectrum of the “chemical environment” (Rappaport and Smith 2010), in interpreting results from the omics assays, and in evaluating the potential contributions of nongenetic variability factors.

At these lower tiers, a probability distribution may best acknowledge the many uncertainties involved in making inferences with limited data. Systematic analyses of chemical sets will be needed to refine distributions for the chemical-specific and general case. For instance, external comparisons of *in vitro* measures based on genetic variability in pharmacodynamics to *in vivo* observations may inform the choice of distribution used for a particular chemical or chemical category. Standard categories, comprising different size and variance distributions for multiple variability factors that can then be applied to other chemicals, may emerge from these analyses. The ranking and grouping of chemicals for the application of these distributions may be based on structural class, the relative extent of observed variability, a common determinant of variability (e.g., as identified in GWAS analysis of cytotoxicity phenotypes), or other factors (e.g., likelihood of coexposures or confounders). Compounds demonstrated or predicted to have highly variable toxic responses may also be given a higher priority for further study, in combination with chemical and other expected modifiers of susceptibility.

At higher tiers of NexGen human health risk assessments, animal and in some cases human data are available for evaluating dose–response relationships, major pathways for some of the critical toxicities for risk assessment can be reasonably well understood, and some *in vivo* human data relevant to those pathways may be available. For some chemicals, sensitive populations may have been identified and studied using omics technologies. In the case of ozone, for example, gene expression data and genomic markers may be collected on individuals of high and average sensitivity. Toxicity pathways exhibited in cultured airway epithelial cells exposed to ozone may also be compared with those in humans exposed *in vivo* to ozone. Such data will aid a better characterization of the dose–time–response severity relationships at low doses. In other cases, where individuals are studied epidemiologically, the current bioinformatics analyses lack power and require pooling of subjects to detect trends, losing variability estimation in the process. In such cases, there will be a need to couple default descriptions of PD variability with PBPK modeling to obtain an overall prediction of variability. In the future, new hypothesis-based molecular clinical and epidemiological approaches that integrate emerging biological knowledge of pathways with observations of physiological disease status, markers of early biological response, and genetics are likely to provide the way forward with population-based descriptions of variability.

## Conclusions

Emerging data streams can inform multiple aspects of biological variability, be used in different modeling approaches addressing PK and/or PD variability, and have application across different chemical screening and evaluation schemes. Successful examples of addressing PK variability include the development and application of a Bayesian PBPK model–based analysis systematically estimating model parameters and characterizing their uncertainty and variability for TCE, a chemical with complex toxicokinetics (Chiu et al. 2009). Additionally, data from animal models and large-scale *in vitro* screening platforms that have incorporated population-based genetic determinants (reviewed by Rusyn et al. 2010), have provided insight into the extent of genetic variability in response to a diversity of toxicants, as well as aided in the identification of genetic susceptibility factors that underscore the development of toxic phenotypes. Hypothesis-based molecular clinical and epidemiological approaches to integrating genetics, molecular pathway data, and clinical observations and biomarkers are likely to contribute to population-based descriptions of variability. Complementary to these are genome-wide (Hutter et al. 2012) and exposure-wide (Patel et al. 2010) approaches for assessing human population variability in toxic response. Opportunities exist to employ these emerging data streams in the development of *in silico* predictive models for application in a range of decision-making contexts.

## References

[r1] Allen BC, Hack CE, Clewell HJ (2007). Use of Markov Chain Monte Carlo analysis with a physiologically-based pharmacokinetic model of methylmercury to estimate exposures in US women of childbearing age.. Risk Anal.

[r2] Beal SL, Sheiner LB (1982). Estimating population kinetics.. Crit Rev Biomed Eng.

[r3] Bernillon P, Bois FY (2000). Statistical issues in toxicokinetic modeling: a Bayesian perspective.. Environ Health Perspect.

[r4] Bois FY (2010). Physiologically based modelling and prediction of drug interactions.. Basic Clin Pharmacol.

[r5] Bois FY, Compton-Quintana PJ (1992). Sensitivity analysis of a new model of carcinogenesis.. J Theor Biol.

[r6] Bois FY, Gelman A, Jiang J, Maszle DR, Zeise L, Alexeef G (1996). Population toxicokinetics of tetrachloroethylene.. Arch Toxicol.

[r7] Bois FY, Jamei M, Clewell HJ (2010). PBPK modelling of inter-individual variability in the pharmacokinetics of environmental chemicals.. Toxicology.

[r8] Bois FY, Krowech G, Zeise L (1995). Modeling human interindividual variability in metabolism and risk: the example of 4-aminobiphenyl.. Risk Anal.

[r9] Bois FY, Zeise L, Tozer TN (1990). Precision and sensitivity of pharmacokinetic models for cancer risk assessment: tetrachloroethylene in mice, rats, and humans.. Toxicol Appl Pharmacol.

[r10] Bradford BU, Lock EF, Kosyk O, Kim S, Uehara T, Harbourt D (2011). Interstrain differences in the liver effects of trichloroethylene in a multistrain panel of inbred mice.. Toxicol Sci.

[r11] Brown JS, Bateson TF, McDonnell WF (2008). Effects of exposure to 0.06 ppm ozone on FEV_1_ in humans: a secondary analysis of existing data.. Environ Health Perspect.

[r12] Burmaster DE, Murray DM (1998). A trivariate distribution for the height, weight, and fat of adult men.. Risk Anal.

[r13] Chen Y, Zhu J, Lum PY, Yang X, Pinto S, MacNeil DJ (2008). Variations in DNA elucidate molecular networks that cause disease.. Nature.

[r14] Chiu WA Euling SY Scott CS Subramaniam RP 2010 Approaches to advancing quantitative human health risk assessment of environmental chemicals in the post-genomic era. Toxicol Appl Pharmacol; doi.org/10.1016/j.taap.2010.03.019 [Online 29 March 2010] 10.1016/j.taap.2010.03.019 20353796

[r15] Chiu WA, Okino MS, Evans MV (2009). Characterizing uncertainty and population variability in the toxicokinetics of trichloroethylene and metabolites in mice, rats, and humans using an updated database, physiologically based pharmacokinetic (PBPK) model, and Bayesian approach.. Toxicol Appl Pharmacol.

[r16] Collins FS, Gray GM, Bucher JR (2008). Transforming environmental health protection.. Science.

[r17] Crump KS, Chen C, Chiu WA, Louis TA, Portier CJ, Subramaniam RP (2010). What role for biologically based dose–response models in estimating low-dose risk?. Environ Health Perspect.

[r18] Csajka C, Verotta D. (2006). Pharmacokinetic–pharmacodynamic modelling: history and perspectives.. J Pharmacokinet Pharmacodyn.

[r19] Dorne JL (2010). Metabolism, variability and risk assessment.. Toxicology.

[r20] Dumas ME, Wilder SP, Bihoreau MT, Barton RH, Fearnside JF, Argoud K (2007). Direct quantitative trait locus mapping of mammalian metabolic phenotypes in diabetic and normoglycemic rat models.. Nat Genet.

[r21] Durbin R, Abecasis G, Altshuler D, Auton A, Brooks L, Gibbs R. (2010). A map of human genome variation from population-scale sequencing.. Nature.

[r22] Emilsson V, Tholeifsson G, Zhang B, Leonardson AS, Zink F, Zhu J (2008). Genetics of gene expression and its effect on disease.. Nature.

[r23] Gieger C Geistlinger L Altmaier E de Angelis MH Kronenberg F Meitinger T 2008 Genetics meets metabolomics: a genome-wide association study of metabolite profiles in human serum. PLoS Genet 4 11 e1000282; doi:10.1371/journal.pgen.1000282 [Online 28 November 2008] 19043545 PMC2581785

[r24] Ginsberg G, Angle K, Guyton K, Sonawane B. (2011). Polymorphism in the DNA repair enzyme XRCC1: utility of current database and implications for human health risk assessment.. Mutat Res.

[r25] Ginsberg G, Guyton K, Johns D, Schimek J, Angle K, Sonawane B. (2010). Genetic polymorphism in metabolism and host defense enzymes: implications for human health risk assessment.. Crit Rev Toxicol.

[r26] Ginsberg G, Hattis D, Sonawane B. (2004). Incorporating pharmacokinetic differences between children and adults in assessing children’s risks to environmental toxicants.. Toxicol Appl Pharmacol.

[r27] Ginsberg G, Hattis D, Sonawane B, Russ A, Banati P, Kozlak M (2002). Evaluation of child/adult pharmacokinetic differences from a database derived from the therapeutic drug literature.. Toxicol Sci.

[r28] Ginsberg G, Neafsey P, Hattis D, Guyton KZ, Johns DO, Sonawane B (2009a). Genetic polymorphism in paraoxonase 1 (PON1): Population distribution of PON1 activity.. J Toxicol Environ Health B Crit Rev.

[r29] Ginsberg G, Smolenski S, Hattis D, Guyton KZ, Johns DO, Sonawane B (2009b). Genetic polymorphism in glutathione transferases (GST): population distribution of GSTM1, T1, and P1 conjugating activity.. J Toxicol Environ Health B Crit Rev.

[r30] Ginsberg G, Smolenski S, Neafsey P, Hattis D, Walker K, Guyton KZ (2009c). The influence of genetic polymorphisms on population variability in six xenobiotic-metabolizing enzymes.. J Toxicol Environ Health B Crit Rev.

[r31] Giorgino T, D’Abramo M, Gervasio F, De Fabritiis G (2010). Exploring the kinetics of drug binding to the hERG channel through large-scale simulations [Abstract]. In: Proceedings of the 2010 Virtual Physiological Human Conference, 30 September—1 October 2010, pp. 248–250. Bruxelles, Belgium.. http://www.vph-noe.eu/vph-repository/doc_download/204-book-of-abstracts-for-vph20107.

[r32] Guo Y, Lu P, Farrell E, Zhang X, Weller P, Monshouwer M (2007). *In silico and in vitro* pharmacogenetic analysis in mice.. Proc Natl Acad Sci USA.

[r33] Guo Y, Weller P, Farrell E, Cheung P, Fitch B, Clark D (2006). *In silico* pharmacogenetics of warfarin metabolism.. Nat Biotechnol.

[r34] Guyton KZ, Kyle AD, Aubrecht J, Cogliano VJ, Eastmond DA, Jackson M (2009). Improving prediction of chemical carcinogenicity by considering multiple mechanisms and applying toxicogenomic approaches.. Mutat Res Rev Mutat Res.

[r35] Hack CE (2006). Bayesian analysis of physiologically based toxicokinetic and toxicodynamic models.. Toxicology.

[r36] Hamade AK, Misra V, Rabold R, Tankersley CG (2010). Age-related changes in cardiac and respiratory adaptation to acute ozone and carbon black exposures: interstrain variation in mice.. Inhal Toxicol.

[r37] Harrill AH, Ross PK, Gatti DM, Threadgill DW, Rusyn I (2009a). Population-based discovery of toxicogenomics biomarkers for hepatotoxicity using a laboratory strain diversity panel.. Toxicol Sci.

[r38] Harrill AH, Watkins PB, Su S, Ross PK, Harbourt DE, Stylianou IM (2009b). Mouse population-guided resequencing reveals that variants in CD44 contribute to acetaminophen-induced liver injury in humans.. Genome Res.

[r39] Hattis D. (1997). Human variability in susceptibility: how big, how often, for what responses to what agents?. Environ Toxicol Pharmacol.

[r40] Hattis D, Baird S, Goble R. (2002). A straw man proposal for a quantitative definition of the RfD.. Drug Chem Toxicol.

[r41] Hattis D, Ginsberg G, Sonawane B, Smolenski S, Russ A, Kozlak M (2003). Differences in pharmacokinetics between children and adults: II. Childrens variability in drug elimination half-lives and in some parameters needed for physiologically-based pharmacokinetic modeling.. Risk Anal.

[r42] Hattis D, Russ A, Goble R, Banati P, Chu M. (2001). Human interindividual variability in susceptibility to airborne particles.. Risk Anal.

[r43] Hernandez ML, Lay JC, Harris B, Esther CR Jr, Brickey WJ, Bromberg PA, et al 2010 Atopic asthmatic subjects but not atopic subjects without asthma have enhanced inflammatory response to ozone. J Allergy Clin Immunol 126(3):537–544.e1; doi:10.1016/j.jaci.2010.06.043 PMC2949284 20816188

[r44] Holloway JW, Savarimuthu Francis S, Fong KM, Yang IA (2012). Genomics and the respiratory effects of air pollution exposure.. Respirology.

[r45] Huang RS, Johnatty SE, Gamazon ER, Im HK, Ziliak D, Duan S (2011). Platinum sensitivity-related germline polymorphism discovered via a cell-based approach and analysis of its association with outcome in ovarian cancer patients.. Clin Cancer Res.

[r46] Hucka M, Finney A, Sauro HM, Bolouri H, Doyle JC, Kitano H (2003). The systems biology markup language (SBML): a medium for representation and exchange of biochemical network models.. Bioinformatics.

[r47] Hutter CM, Chang-Claude J, Slattery ML, Pflugeisen BM, Lin Y, Duggan D (2012). Characterization of gene–environment interactions for colorectal cancer susceptibility loci.. Cancer Res.

[r48] Illig T, Gieger C, Zhai G, Römisch-Margl W, Wang-Sattler R, Prehn C (2010). A genome-wide perspective of genetic variation in human metabolism.. Nat Genet.

[r49] International Programme for Chemical Safety (2001). Guidance Document for the Use of Data in Development of Chemical-Specific Adjustment Factors (CSAFs) for Interspecies Differences and Human Variability in Dose/Concentration–Response Assessment. WHO/PCS/01.4.. http://www.who.int/ipcs/publications/methods/harmonization/en/csafs_guidance_doc.pdf.

[r50] Jamei M, Marciniak S, Feng K, Barnett A, Tucker G, Rostami-Hodjegan A. (2009). The Simcyp population-based ADME simulator.. Expert Opin Drug Metab Toxicol.

[r51] Jia X, Song X, Shima M, Tamura K, Deng F, Guo X. (2011). Acute effect of ambient ozone on heart rate variability in healthy elderly subjects.. J Expo Sci Environ Epidemiol.

[r52] Johanson G, Jonsson F, Bois F. (1999). Development of new technique for risk assessment using physiologically based toxicokinetic models.. Am J Ind Med.

[r53] Jonsson F, Jonsson EN, Bois FY, Marshall S (2007). The application of a Bayesian approach to the analysis of a complex, mechanistically based model.. J Biopharm Stat.

[r54] Judson RS, Kavlock RJ, Setzer RW, Hubal EA, Martin MT, Knudsen TB (2011). Estimating toxicity-related biological pathway altering doses for high-throughput chemical risk assessment.. Chem Res Toxicol.

[r55] Kedderis G. (1997). Extrapolation of *in vitro* enzyme induction data to humans *in vivo*.. Chem Biol Interact.

[r56] Khoury MJ, Bowen MS, Burke W, Coates RJ, Dowling NF, Evans JP (2011). Current priorities for public health practice in addressing the role of human genomics in improving population health.. Am J Prev Med.

[r57] Kleeberger SR, Levitt RC, Zhang LY, Longphre M, Harkema J, Jedlicka A (1997). Linkage analysis of susceptibility to ozone-induced lung inflammation in inbred mice.. Nat Genet.

[r58] Koturbash I, Scherhag A, Sorrentino J, Sexton K, Bodnar W, Swenberg JA (2011). Epigenetic mechanisms of mouse interstrain variability in genotoxicity of the environmental toxicant 1,3-butadiene.. Toxicol Sci.

[r59] La Thangue NB, Kerr DJ (2011). Predictive biomarkers: a paradigm shift towards personalized cancer medicine.. Nat Rev Clin Oncol.

[r60] Lock EF, Abdo N, Huang R, Xia M, Kosyk O, O’Shea SH (2012). Quantitative high-throughput screening for chemical toxicity in a population-based *in vitro* model.. Toxicol Sci.

[r61] Luke NS, Devito MJ, Shah I, El-Masri HA (2010a). Development of a quantitative model of pregnane X receptor (PXR) mediated xenobiotic metabolizing enzyme induction.. Bull Math Biol.

[r62] Luke NS, Sams R, DeVito MJ, Conolly RB, El-Masri HA (2010b). Development of a quantitative model incorporating key events in a hepatotoxic mode of action to predict tumor incidence.. Toxicol Sci.

[r63] Manolio TA (2010). Genomewide association studies and assessment of the risk of disease.. N Engl J Med.

[r64] Manolio TA, Collins FS, Cox NJ, Goldstein DB, Hindorff LA, Hunter DJ (2009). Finding the missing heritability of complex diseases.. Nature.

[r65] Martin MT, Dix DJ, Judson RS, Kavlock RJ, Reif DM, Richard AM (2010). Impact of environmental chemicals on key transcription regulators and correlation to toxicity end points within EPA’s ToxCast program.. Chem Res Toxicol.

[r66] McLanahan ED, Andersen ME, Fisher JW (2008). A biologically based dose-response model for dietary iodide and the hypothalamic-pituitary-thyroid axis in the adult rat: evaluation of iodide deficiency.. Toxicol Sci.

[r67] Mortensen HM Euling SY 2011 Integrating mechanistic and polymorphism data to characterize human genetic susceptibility for environmental chemical risk assessment in the 21st century. Toxicol Appl Pharmacol; doi:10.1016/j.taap.2011.01.015 [Online 1 February 2011] 21291902

[r68] Neafsey P, Ginsberg G, Hattis D, Johns DO, Guyton KZ, Sonawane B (2009a). Genetic polymorphism in CYP2E1: Population distribution of CYP2E1 activity.. J Toxicol Environ Health B Crit Rev.

[r69] Neafsey P, Ginsberg G, Hattis D, Sonawane B. (2009b). Genetic polymorphism in cytochrome P450 2D6 (CYP2D6): Population distribution of CYP2D6 activity.. J Toxicol Environ Health B Crit Rev.

[r70] Nong A, Tan YM, Krolski ME, Wang J, Lunchick C, Conolly RB (2008). Bayesian calibration of a physiologically based pharmacokinetic/pharmacodynamic model of carbaryl cholinesterase inhibition.. J Toxicol Environ Health A.

[r71] NRC (National Research Council) (2007). Toxicity Testing in the 21st Century: A Vision and a Strategy. Washington, DC:National Academies Press.. http://www.nap.edu/catalog.php?record_id=11970.

[r72] NRC (National Research Council) (2009). Science and Decisions: Advancing Risk Assessment. Washington, DC:National Academies Press.. http://www.nap.edu/catalog.php?record_id=12209.

[r73] O’Shea SH, Schwarz J, Kosyk O, Ross PK, Ha MJ, Wright FA (2011). *In vitro* screening for population variability in chemical toxicity.. Toxicol Sci.

[r74] Patel CJ Bhattacharya J Butte AJ 2010 An Environment-Wide Association Study (EWAS) on type 2 diabetes mellitus. PLoS ONE 5 5 e10746; doi:10.1371/journal.pone.0010746 [Online 20 May 2010] 20505766 PMC2873978

[r75] Peters EJ, Motsinger-Reif A, Havener TM, Everitt L, Hardison NE, Watson VG (2011). Pharmacogenomic characterization of US FDA-approved cytotoxic drugs.. Pharmacogenomics.

[r76] Phillips EJ, Mallal SA (2010). Pharmacogenetics of drug hypersensitivity.. Pharmacogenomics.

[r77] Portier CJ, Kaplan NL (1989). Variability of safe dose estimates when using complicated models of the carcinogenic process. A case study: methylene chloride.. Fundam Appl Toxicol.

[r78] Prows DR, Shertzer HG, Daly MJ, Sidman CL, Leikauf GD (1997). Genetic analysis of ozone-induced acute lung injury in sensitive and resistant strains of mice.. Nat Genet.

[r79] Rappaport SM, Smith MT (2010). Epidemiology. Environment and disease risks.. Science.

[r80] Reif DM, Martin MT, Tan SW, Houck KA, Judson RS, Richard AM (2010). Endocrine profiling and prioritization of environmental chemicals using ToxCast data.. Environ Health Perspect.

[r81] Rosenberg NA, Huang L, Jewett EM, Szpiech ZA, Jankovic I, Boehnke M (2010). Genome-wide association studies in diverse populations.. Nat Rev Genet.

[r82] Rotroff DM, Wetmore BA, Dix DJ, Ferguson SS, Clewell HJ, Houck KA (2010). Incorporating human dosimetry and exposure into high-throughput *in vitro* toxicity screening.. Toxicol Sci.

[r83] Roy AC, Loke DF, Saha N, Viegas OA, Tay JS, Ratnam SS (1994). Interrelationships of serum paraoxonase, serum lipids and apolipoproteins in normal pregnancy. A longitudinal study.. Gynecol Obstet Invest.

[r84] Rusyn I, Gatti DM, Wiltshire T, Wiltshire T, Kleeberger SR, Threadgill DW (2010). Toxicogenetics: population-based testing of drug and chemical safety in mouse models.. Pharmacogenomics.

[r85] Sadiq SK, Wright DW, Kenway OA, Coveney PV (2010). Accurate ensemble molecular dynamics binding free energy ranking of multidrug-resistant HIV-1 proteases.. J Chem Inf Model.

[r86] Schadt EE (2009). Molecular networks as sensors and drivers of common human diseases.. Nature.

[r87] Schadt EE, Björkegren JL 2012 NEW: network-enabled wisdom in biology, medicine, and health care. Sci Transl Med 4(115):115rv111; doi:10.1126/scitranslmed.3002132 [Online 4 January 2012]. 22218693

[r88] Schuster P 2008 Modeling in biological chemistry. From biochemical kinetics to systems biology. Monatshefte fur Chemie / Chemical Monthly 139(4):427–446.

[r89] Spear RC, Bois FY (1994). Parameter variability and the interpretation of physiologically based pharmacokinetic modeling results.. Environ Health Perspect.

[r90] Suhre K, Shin SY, Petersen AK, Mohney RP, Meredith D, Wägele B (2011). Human metabolic individuality in biomedical and pharmaceutical research.. Nature.

[r91] Thompson CM, Johns DO, Sonawane B, Barton HA, Hattis D, Tardif R (2009). Database for physiologically based pharmacokinetic (PBPK) modeling: physiological data for healthy and health-impaired elderly.. J Toxicol Environ Health B Crit Rev.

[r92] U.S. EPA (U.S. Environmental Protection Agency) (1998). Toxicological Review of Beryllium and Compounds (CAS No.7440-41-7) in Support of Summary Information on the Integrated Risk Information System (IRIS). EPA/635/R-98/008. Washington, DC:U.S. EPA.. http://www.epa.gov/iris/toxreviews/0012tr.pdf.

[r93] U.S. EPA (U.S. Environmental Protection Agency) (2002). Health Assessment of 1,3-Butadiene. EPA/600/P-98/001F. Washington, DC:U.S. EPA, Office of Research and Development, National Center for Environmental Assessment.. http://cfpub.epa.gov/ncea/cfm/recordisplay.cfm?deid=54499.

[r94] U.S. EPA (U.S. Environmental Protection Agency) (2003). EPA Assessment of Risks from Radon in Homes. EPA/402/R-03/003. Washington, DC:U.S. EPA.. http://www.epa.gov/radiation/docs/assessment/402-r-03-003.pdf.

[r95] U.S. EPA (U.S. Environmental Protection Agency) (2006). Air Quality Criteria for Ozone and Related Photochemical Oxidants. EPA/600/R-05/004aF-cF. Washington, DC:U.S. EPA.. http://cfpub.epa.gov/ncea/isa/recordisplay.cfm?deid=149923.

[r96] U.S. EPA (U.S. Environmental Protection Agency) (2007). Framework for Assessing the Public Health Impacts of Risk Management Decisions.

[r97] U.S. EPA (U.S. Environmental Protection Agency) (2011). Toxicological Review of Trichloroethylene (CASRN 79-01-6) in Support of Summary Information on the Integrated Risk Information System (IRIS). EPA/635/R-09/011F. Washington, DC:U.S. EPA.. http://www.epa.gov/iris/supdocs/0199index.html.

[r98] Wakefield J. (1996). Bayesian individualization via sampling-based methods.. J Pharmacokinet Biopharm.

[r99] Walker K, Ginsberg G, Hattis D, Johns D, Guyton KZ, Sonawane B (2009). Genetic polymorphism in *N*-acetyltransferase (NAT): population distribution of NAT1 and NAT2 activity.. J Toxicol Environ Health B Crit Rev.

[r100] Watson VG Motsinger-Reif A Hardison NE Peters EJ Havener TM Everitt L 2011 Identification and replication of loci involved in camptothecin-induced cytotoxicity using CEPH pedigrees. PloS one 6 5 e17561; doi:10.1371/journal.pone.0017561 [Online 5 May 2011] 21573211 PMC3088663

[r101] Welsh M, Mangravite L, Medina MW, Tantisira K, Zhang W, Huang RS (2009). Pharmacogenomic discovery using cell-based models.. Pharmacol Rev.

[r102] Wild CP (2005). Complementing the genome with an “exposome:” the outstanding challenge of environmental exposure measurement in molecular epidemiology.. Cancer Epidemiol Biomarkers Prev.

[r103] Willmann S, Höhn K, Edginton A, Sevestre M, Solodenko J, Weiss W (2007). Development of a physiology-based whole-body population model for assessing the influence of individual variability on the pharmacokinetics of drugs.. J Pharmacokinet Pharmacodyn.

[r104] Wood AM, Harrison RM, Semple S, Ayres JG, Stockley RA (2010). Outdoor air pollution is associated with rapid decline of lung function in α-1-antitrypsin deficiency.. Occup Environ Med.

[r105] Woodruff TJ, Zeise L, Axelrad DA, Guyton KZ, Janssen S, Miller M (2008). Meeting report: Moving upstream—evaluating adverse upstream end points for improved risk assessment and decision-making.. Environ Health Perspect.

[r106] Xia M, Huang R, Witt KL, Southall N, Fostel J, Cho MH (2008). Compound cytotoxicity profiling using quantitative high-throughput screening.. Environ Health Perspect.

[r107] Xia MH, Huang RL, Guo V, Southall N, Cho MH, Inglese J (2009). Identification of compounds that potentiate CREB signaling as possible enhancers of long-term memory.. Proc Natl Acad Sci USA.

[r108] Zhu H, Rusyn I, Richard A, Tropsha A. (2008). Use of cell viability assay data improves the prediction accuracy of conventional quantitative structure–activity relationship models of animal carcinogenicity.. Environ Health Perspect.

